# Evaluation of three novel antigens and costimulatory agents for improvement of *M. Tuberculosis* specific interferon gamma release assays

**DOI:** 10.1186/s12879-025-10577-3

**Published:** 2025-02-07

**Authors:** Sandra Schwarzlose-Schwarck, Mark Reinwald, Torsten Bauer, Florian Hentschel, Til Kiderlen, Dorinja Zapf, Victor Herbst, Stefan Lüth, David Krieger, Werner Dammermann

**Affiliations:** 1 Department of Hematology and Oncology, Brandenburg Medical School Theodor Fontane, University Hospital Brandenburg, Brandenburg an der Havel, Germany; 2 Respiratory Diseases Clinic, Heckeshorn, Helios Klinikum Emil von Behring, Berlin, Germany; 3 Department of Gastroenterology, University Hospital Brandenburg, Brandenburg Medical School Theodor Fontane, Hochstrasse 29, 14770 Brandenburg, Germany; 4 Institute for Experimental Immunology, Affiliated to EUROIMMUN Medizinische Labordiagnostika AG, 23560 Lübeck, Germany; 5 Faculty of Health Sciences Brandenburg, Brandenburg Medical School Theodor Fontane, Brandenburg an der Havel, Germany; 6 Center of Translational Medicine, Brandenburg Medical School Theodor Fontane, Brandenburg an der Havel, Germany; 7 Department of Oncology, Vivantes Auguste-Viktoria-Klinikum, Berlin, Germany

**Keywords:** Tuberculosis, IGRA, Complement factors, TLR agonists, Interferon gamma release assay

## Abstract

**Background:**

*Mycobacterium tuberculosis* (MT) infections represent a global health problem and latent tuberculosis infection (LTBI) affects an estimated 25% of the world population. 10.6 million people fell ill with tuberculosis (TB) worldwide in 2021 and a total of 1.6 million TB-associated deaths were reported. Thus, reliable diagnosis of LTBI is crucial to ensure adequate treatment. We tested three novel MT antigens of the dormancy survival regulator (DosR) complex, ACR, Rv1733, Rv2626, for improvement of MT specific interferon gamma release assays (IGRA) for diagnosing TB. Furthermore, we specifically investigated the potential of the complement factor C5a and the toll like receptor (TLR) agonists CpG ODN as well as Poly(I: C) as costimulators in order to increase diagnostic quality of MT IGRAs. Three MT IGRAs were evaluated, i.e. our in-house IGRA, a prototypic EUROIMMUN Quan-T-Cell TB assay and the gold standard QuantiFERON Tb-Gold Plus assay.

**Methods:**

*In this single-center*,* prospective trial*, whole blood from 71 patients with tuberculosis disease was stimulated using our in-house IGRA with ACR, Rv1733, Rv2626 compared to the current gold standard MT antigen formulation encompassing MT antigens ESAT-6, CFP-10 and TB10.4. Further, C5a, CpG ODN and Poly(I: C) were tested as co-stimulators. IFN-γ levels in plasma were quantified using ELISA.

**Results:**

The three novel antigens ACR, Rv1733 and Rv2626 failed to elicit equal or stronger IFN-γ-responses compared to the gold standard antigen formulation with ESAT-6, CFP-10 and TB10.4. The TLR9 agonist CpG ODN increased IFN-γ responses in whole blood of tuberculosis patients using our in-house assays (6,768 ± 21,097 mlU/ml vs. 2,971 ± 4,780 mlU/ml, *p* = 0.31), yet not significantly. The same trend was found for the prototypic EUROIMMUN Quan-T-Cell TB assay (3,355 ± 5,425 mlU/ml vs. 2,548 ± 4,145 mlU/ml, *p* = 0.1) and the QuantiFERON Tb-Gold Plus assay (3,627 ± 5,992 mlU/ml vs. 2,635 m ± 4,475 mlU/ml, *p* = 0.08, for tube 1; 3,257 ± 5,349 vs. 2,759 ± 4,446 mIU/ml, *p* = 0.25, for tube 2). No increase of IFN-γ release was seen using Poly(I: C) or C5a in all three assays.

**Conclusions:**

ACR, Rv1733 and Rv2626 failed to elicit equal or even better IFN-γ responses in our in-house IGRA compared to ESAT-6, CFP-10 and TB10.4 in patients with MT infection. The TLR9 agonist CpG ODN might be useful as co-stimulator in MT IGRAs.

## Introduction

According to World Health Organisation (WHO) data an estimated 10.6 million people world-wide presented with active tuberculosis (TB) in 2021 [1] which represents a serious health burden. Prior to open infection, the majority of these patients are afflicted with a subclinical latent tuberculosis infection (LTBI). LTBI is defined by the WHO as a state of persistent immune response to *Mycobacterium tuberculosis* (MT) antigens without overt clinical symptoms [2]. It is estimated that about 25% of the world’s population carry LTBI [3], of whom 5–10% will convert to active disease [4]. Established risk factors for conversion from LTBI to TB are diverse, e.g. malnutrition, immunocompromising diseases or medication as well as very young or old age [5].

Active TB is diagnosed by identifying MT by culture or polymerase chain reaction (PCR) in clinical samples such as sputum or tissue whereas LTBI is diagnosed by two major in vitro diagnostic (IVD) tests [6]. The conventional and still frequently used test is the tuberculin skin test (TST). Tuberculin is a standardised inactivated extract of cultured MT injected intracutaneously in order to assess the immune response to MT. If positive, an inflammatory reaction appears at the site of injection after several hours, which then can be assessed by a trained physician. TST is in widespread use, can be performed anywhere and carries low costs, but is limited by numerous factors: Leading drawbacks are a low specificity, errors due to false application of tuberculin or false interpretation of the immune reaction by untrained physicians; in addition, factors such as immunodeficiency may decrease reactivity [7]. Further limitations are false positive reactions due to prior immunization with Bacille Calmette-Guérin (BCG), a vaccine based on attenuated *Mycobacterium bovis* [8]. Notably, an immune booster effect can occur after multiple TST uses in a single individual leading to false positive results. Lot-to-lot differences in tuberculin quality, i.e. purity, yield, can also have a documented negative impact on TST quality, i.e. specificity and sensitivity.

The second IVD test type available for diagnosis of LTBI are serologic IFN-γ release assays (IGRA) which measure the cellular immune host response to stimulatory antigens. The ELISA-based QuantiFERON-TB-Gold measures the amount of IFN-γ released by T-cells in whole blood after antigen challenge, while the ELISPOT-based T-SPOT.TB assay quantifies the absolute number of T-cells that produce IFN-γ after antigen challenge [6, 9, 10]. Of note, several more commercial IVD tests have been introduced by now, e.g. TB-IGRA, LIOFeron TB/LTBI, CLIA-IGRA [11–13]. The two specific antigens of MT used in the most prominent assays, QuantiFERON-TB-Gold and T-SPOT-TB assay, are the 6 kDa early-secreted antigenic target protein (ESAT-6) and the 10 kDa culture filtrate protein (CFP-10), which are not present in BCG or *Mycobacteria other than tuberculosis* (MOTT). Advantages of IGRA assays comprise the higher specificity, standardisation and objectivity of the procedure and the performance including a positive and a negative control. In addition, there is no risk of immune boosting the result.

The diagnostic performance and predictive potential of both test types, IGRA and TST, is still under discussion: For example, Rangaka and colleagues [14] found that IGRA and TST predict the same incidence risk ratio (RR) for risk of conversion of LTBI to overt active tuberculosis while Zhou et al. [15] found the IGRAs to have a significantly higher RR-prediction than TST.

Established IGRAs used for diagnosis of latent MT infection have however limited sensitivity [16]. Thus, alternative MT latency-associated antigens or costimulatory molecules may improve diagnostic performance of these assays which represents a clinical need.

Therefore, the main objectives of our study were to evaluate new potent antigens of MT, i.e. ACR, Rv1733, Rv2626, in order to improve diagnostic performance of IGRA for MT. These classes of antigens belong to the dormancy survival regulator (DosR), which mediates MT latency [17]. These latency antigens may potentially induce a strong MT specific immune response and thus form a memory T cell population preceding later TB infection. Including antigens from both latent as well as active TB disease might increase the diagnostic quality of MT specific IGRAs. In addition, we aimed at mimicking the proinflammatory microenvironment associated with latent TB infection, in order to enhance the diagnostic quality of the IGRA test platform. We specifically investigated the potential of complement factors as well as toll like receptor (TLR) agonists to boost IFN-γ responses in IGRAs.

We tested the complement factors C5a and the TLR agonists ODN as well as Poly(I: C) which we recently introduced as potential costimulators to our own in-house IGRA test platform in order to increase the diagnostic sensitivity and specificity [18–20].

By recruiting a cohort of patients with diagnosed TB infection we could assess diagnostic quality in direct comparison to the gold standard IGRA, the QuantiFERON Tb-Gold Plus test.

## Methods

### Patient selection

Patients with tuberculosis disease treated at the tuberculosis department of the Helios Hospital Emil von Behring, Berlin, were enrolled in the study for which all patients gave informed consent and which was approved by the Ethics Committee of the Brandenburg Chamber of Physicians (S20(a)/2016) as well as the Berlin Chamber of Physicians (Eth-V-ZK/17). Seventy one patients were recruited (Table 1). MT infection was bacteriologically confirmed or clinically diagnosed.

**Table 1:** Characteristics of all subjects included in the study

Characteristic		
		TB patients
n^b^		71
	Male	56 (79%)
	Female	15 (21%)
Age (yr)^a^		39.4 ± 16.7
	Male	37.9 ± 15.5
	Female	44.2 ± 19.9
Chest radiography		
	Positive	29 (41%)
	Negative	2 (3%)
	Unknown	40 (56%)
Histology		
	Positive	4 (6%)
	Negative	11 (15%)
	Unknown	56 (79%)
PCR		
	Positive	20 (28%)
	Negative	14 (20%)
	Unknown	37 (52%)
Culture		
	Positive	15 (21%)
	Negative	10 (14%)
	Unknown	46 (65%)
Treatment		
	Positive	43 (61%)
	Negative	24 (34%)
	Unknown	4 (5%)

^a^ The data are shown as means ± standard deviations

Thirty one patients with rheumatological diseases and no history of tuberculosis disease or LTBI were enrolled in the control group.

### Reagents

The MT peptide pools ACR, Rv1733, Rv2626c, CFP-10, ESAT-6, TB10.4 were purchased from JPT, Germany (Custom made and #PM-MYCTU-CFP10, PM-MYCTU-ESAT6, PM-MYCTU-TB104) and solved in sterile dimethyl sulfoxide (DMSO; #RO/A9941/000100, Th. Geyer, Germany) to 50 mg/ml followed by storage at -20 °C. As positive control, *Staphylococcus aureus* enterotoxin B superantigen (SEB) was obtained from Sigma Aldrich GmbH, Germany (#S4881) and stored 1 mg/ml in sterile, endotoxin-free H_2_O and − 20 °C. MT peptide libraries of ACR, Rv1733 as well as Rv2626c were custom made by JPT, Germany. Synthetic ACR represented a mix of 34 peptides (15 amino acid length each, 11 aa overlap, peptide scan 15/11) comprising the whole amino acid sequence of Alpha-Crystallin (ACR), a 15.8 kDa MT protein (strain H37Rv, UniProt: P9WMK1) [21, 22]. Synthetic Rv1733c represented a mix of 50 peptides (15 amino acid length each, 11 aa overlap, peptide scan 15/11) comprising the whole amino acid sequence of Rv1733c, a 23.1 kDa MT protein (strain H37Rv, Uniprot: P9WLS9) [23]. Synthetic Rv2626c represented a mix of 33 peptides (15 amino acid length each, 11 aa overlap, peptide scan 15/11) comprising the whole amino acid sequence of Rv2626c, a 15.7 kDa MT protein (strain H37Rv, Uniprot: P9WJA3) [24].

TLR agonists were purchased from InvivoGen, Germany, and reconstituted according to the manufacturer’s technical specifications in sterile H_2_O at 1 mg/ml: Poly(I: C) high molecular weight (#tlrl-pic), ODN2216, ODN2006 and ODN2395 (#tlrl-2216, #tlrl-2006, #tlrl-2395). ODN2216, ODN2006 and ODN2395 were pooled prior to use. All TLR agonists were stored at − 20 °C as one-time-use aliquots. Human recombinant C5a was purchased from Hycult Biotech, Germany (#HC2101). C5a was reconstituted in sterile H_2_O (0.15 mg/ml) and stored at − 20 °C.

### In-house TB IGRA

Venous blood from MT patients was collected in sterile Lithium Heparin Vacutainers (Becton Dickinson, Germany). 0.5 ml of whole blood were then transferred to sterile, pyrogen-free 2 ml tubes (Sarstedt, Germany) and stimulated with MT antigens (10 µg/ml each). Samples stimulated with 0.9% (w/v) NaCl solution served as negative control whereas SEB (1 µg/ml) stimulated samples served as positive controls. Glucose (2 mg/ml final concentration; pre-diluted in sterile 0.9% (w/v) NaCl solution) was added to each tube to further enhance cytokine secretion as described previously [25]. All three TLR9 agonists, ODN2216, ODN2006 and ODN2395, were applied combined at a final concentration of 10 µg/ml each after dilution out of stock. The TLR3 agonist, Poly(I: C), was applied at a final concentration of 1 µg/ml after dilution out of stock. The complement factor C5a was used at a final concentration of 0.75 µg/ml. All tubes were incubated at 37 °C for 24 h. Upon centrifugation plasma supernatants were aspirated, stabilized with 0.045% (w/v) NaN_3_ and stored at − 20 °C until cytokine measurement using the EUROIMMUN Quant-T-Cell ELISA (see Sect. 2.6). The amount of biomaterial per donor was not always sufficient to perform all stimulations which is why for some donor single conditions had to be left out. This explains variations with regard to group size (n).

### EUROIMMUN prototypic Quant-T-cell TB-IGRA

MT specific T cell activity was assessed via stimulation of the T cells using a prototypic EUROIMMUN Quan-T-Cell TB assay that was performed according to the manufacturer’s instructions. In brief, 0.5 ml of heparinized whole blood was transferred into three different tubes (BLANK, TB-Mix and STIM) followed by a 24 h incubation step at 37 °C after inverting these tubes multiple times. The TB-Mix tube was coated with CFP-10 and ESAT-6 antigenic peptides of MT. Following incubation, all tubes were centrifuged and plasma supernatants were aspirated, stabilized with 0.045% (w/v) NaN_3_ and stored at − 20 °C until cytokine measurement using the EUROIMMUN Quant-T-Cell ELISA (see Sect. 2.6).

### QuantiFERON Tb-Gold Plus

In order to assess the diagnostic quality of the in-house TB IGRA as well as the prototypic EUROIMMUN Quan-T-Cell TB assay the current gold standard test was applied, the QuantiFERON Tb-Gold Plus assay. In brief, 1 ml of heparinized whole blood was transferred into four different tubes (NIL, TB1, TB2 and Mitogen) followed by a 24 h incubation step at 37 °C after inverting these tubes multiple times. The TB1 and TB2-tube were coated with CFP-10 as well as ESAT-6 antigenic peptides of MT. Following incubation, all tubes were centrifuged and plasma supernatants were aspirated, stabilized with 0.045% (w/v) NaN_3_ and stored at − 20 °C until cytokine measurement using the EUROIMMUN Quant-T-Cell ELISA (see Sect. 2.6).

### EUROIMMUN Quan-T-Cell TB ELISA for measurement of IFN-γ

Detection of total amounts of IFN-γ in human plasma was conducted using the EUROIMMUN Quant-T-Cell TB ELISA (#EQ 6841–9601) according to the manufacturer’s instructions. Initial dilution of IGRA control and test samples was performed described as follows: negative control 1/5, positive control 1/2500, test samples (MT antigens with/without costimulators) 1/5. A photometric measurement was performed at a wavelength of 450 nm with a reference measurement at 620 nm. If a test sample’s absorbance value fell outside the maximum standard curve range, these samples were subsequently retested with a tenfold higher dilution, e.g. 1/5 ◊ 1/50, 1/500 ◊ 1/5000, 1/2500 ◊ 1/25,000. A six-point standard curve from 0.1 to 333 mIU/ml IFN-γ was used for quantitation. Standards, ELISA controls and test samples were measured in duplicate. Lot-specific lyophilized calibrators and controls included in the assay kit were used as a standard. The samples were analyzed using Magellan software equipped on a Tecan Spark 10 M plate reader.

### Data analysis

#### Software

The ELISA data was analyzed using Graphpad Prism software (Graphpad Software Inc., version number 9.0).

#### Statistical analysis

Descriptive statistics and paired t-tests were performed using and GraphPad Prism software (Graphpad Software Inc., version number 9.0).

## Results

First, viability of all collected samples was confirmed by stimulation with the superantigen SEB, separately. The samples showed a median IFN-γ response of 11,789 mlU/ml (data not shown). Positive control tubes for the prototypic EUROIMMUN Quan-T-Cell TB assay as well as the gold standard QuantiFERON Tb-Gold Plus assay yielded similar results.

### *M. Tuberculosis* dormancy antigens ACR/Rv1733/Rv2626 failed to improve MT IGRA quality

**Fig. 1:**
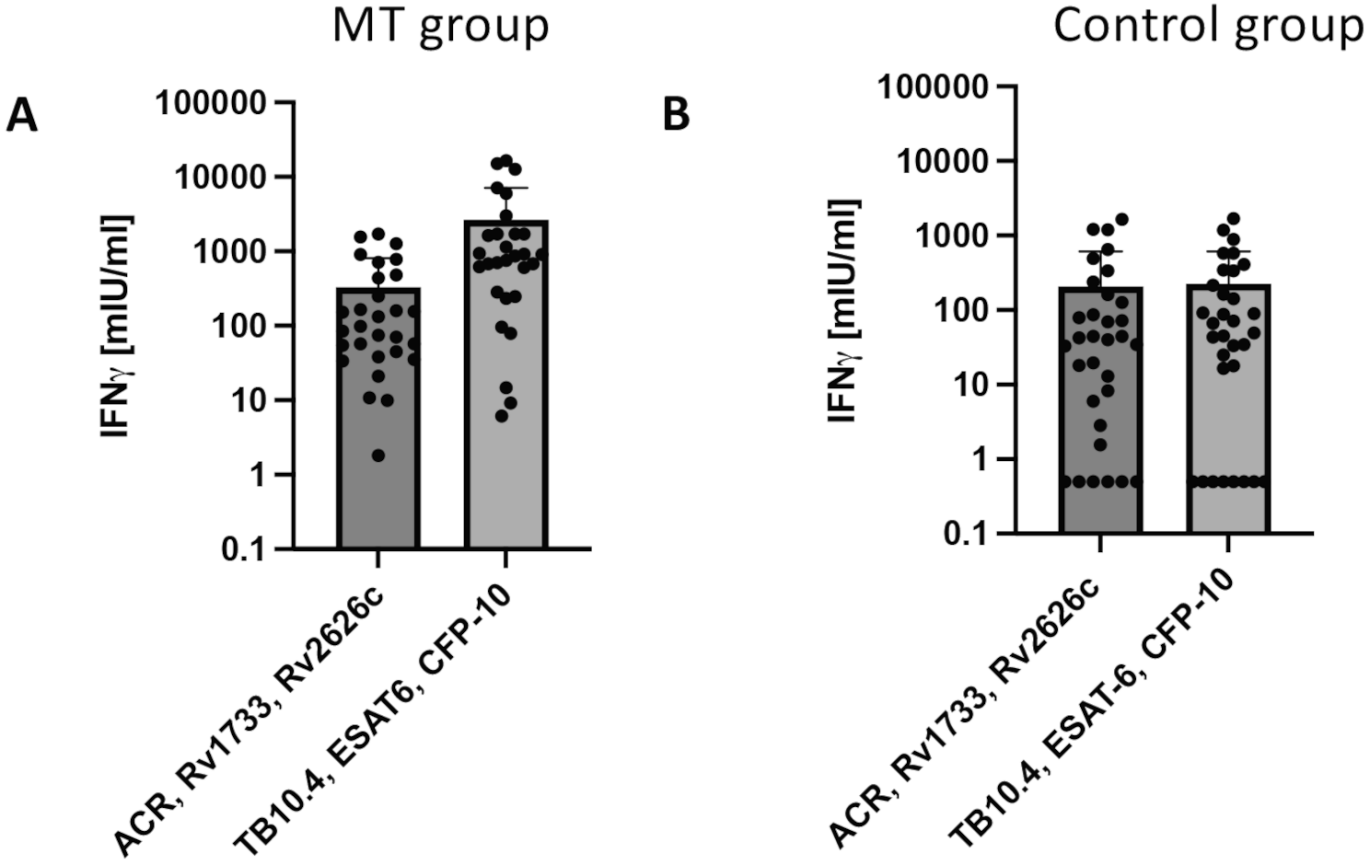
Alternative M. tuberculosis dormancy antigens fail to improve MT IGRA quality. (**A**) IFN-γ responses in whole blood following stimulation with ACR/Rv1733/Rv2626 and TB10.4/ESAT6/CFP10, respectively. (**B**) IFN-γ responses in whole blood following stimulation with ACR/Rv1733/Rv2626 and TB10.4/ESAT6/CFP10 in the control group. All values are given as mean concentration mIU/ml ± S.D. Lower limit of detection (Background + 3x S.D.) was at 1.9–12.6 mIU/ml for IFN-γ. Paired student’s t-test. Following symbols pinpoint significant differences: * *p* < 0.05, ** *p* < 0.01, *** *p* < 0.001 (*n* = 29)

Next, we tested the candidate MT antigens ACR/Rv1733/Rv2626 regarding non-inferior or even superior performance compared to the current gold standard MT antigens TB10.4/ESAT6/CFP10 using our in-house IGRA. After stimulation of whole blood with ACR/Rv1733/Rv2626 the mean production of IFN-γ was significantly lower (329.7 ± 477.6 mlU/ml, *p* = 0.008) compared to the IFN-γ response after stimulation with TB10.4/ESAT6/CFP10 (2,651 ± 4,506 mlU/ml) (Fig. 1A; Table 2). The candidate MT antigens yielded inferior results compared to the gold standard MT antigens.

**Table 2:** MT-specific IFN-γ release using new MT antigens

Assay	In-house MT IGRA^a^	
		IFN-γ [mIU/ml]
Antigen		
	TB10.4/ESAT6/CFP-10	2,651 ± 4,506
	ACR/Rv1733/Rv2626	329.7 ± 477.6
n^b^		29 of 71 total

^a^ The data are shown as means ± standard deviations (S.D.)

^b^ The amount of biomaterial per donor was not always sufficient to perform all stimulations which is why for some donors conditions had to be left out. This explains variations with regard to the group size (*n*)

The IFN-γ responses of the control group (Fig. 1B) were nearly equivalent at the mean after stimulation with ACR/Rv1733/Rv2626 and TB10.4/ESAT6/CFP10 (209.9 ± 412.8 mlU/ml vs.177.9 ± 283.6 mlU/ml), but significantly inferior compared to the IFN-γ response in patients with tuberculosis disease after stimulation with ACR/Rv1733/Rv2626 (209.9 ± 412.8 mlU/ml vs. 329.7 ± 477.6 mlU/ml, *p* = 0.012) and TB10.4/ESAT6/CFP10 (177.9 ± 283.6 mlU/ml vs. 2,651 ± 4,506 mlU/ml, *p* = < 0.0001).

### Effects of TLR agonist and complement factor dependent co-stimulation on IFN-γ responses in three different MT IGRA test systems

Since ACR/Rv1733/Rv2626 failed to elicit equal or even better IFN-γ responses in our in-house IGRA, we aimed at increasing MT IGRA quality by adding TLR agonists or complement factors as co-stimulating reagents. We have shown in previous studies that the TLR agonists CpG ODN and Poly(I: C) as well as the complement factor C5a are able to increase IFN-γ responses in our in-house IGRA significantly. Three different IGRA test systems were included, i.e. our own in-house assay, a prototypic EUROIMMUN Quan-T-Cell TB assay as well as the gold standard QuantiFERON Tb-Gold Plus assay. All assays used gold standard MT antigens, TB10.4/ESAT6/CFP10 for the in-house assay and ESAT6/CFP10 for the QuantiFERON Tb-Gold Plus assay as well as the EUROIMMUN Quan-T-Cell TB assay.

**Fig. 2:**
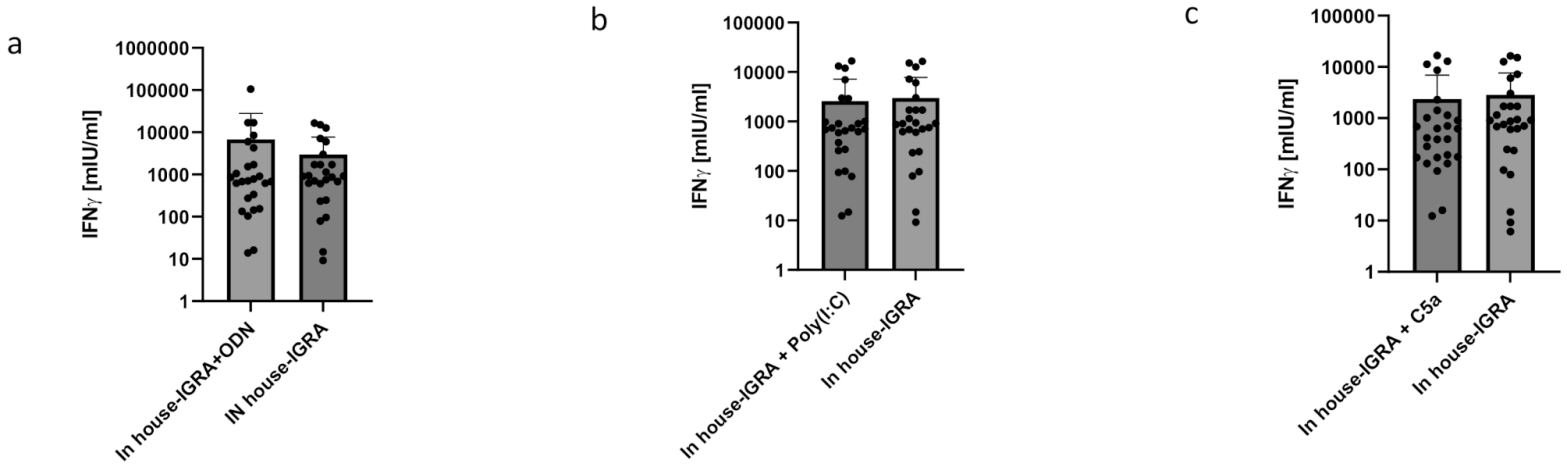
IFN-γ responses with in-house assay (TB10.4/ESAT6/CFP-10) and costimulating factors. No significant increase in IFN-γ was seen with (**a**) TLR9 agonist CpG ODN, (**b**) Poly(I: C) and (**c**) C5a. All values are given as mean concentration mIU/ml ± S.D. Lower limit of detection (Background + 3x S.D.) was at 1.9–12.6 mIU/ml for IFN-γ. Paired student’s t-test. Following symbols pinpoint significant differences: * *p* < 0.05, ** *p* < 0.01, *** *p* < 0.001 (*n* = 25)

Notably, for our in-house assay only the TLR9 agonist CpG ODN increased IFN-γ responses in whole blood of tuberculosis patients, albeit the effect did not reach statistical significance, i.e. 6,768 ± 21,097 mlU/ml vs. 2,971 ± 4,780 mlU/ml, *p* = 0.31 (Fig. 2; Table 3). Addition of Poly(I: C) (2,578 ± 4,579 mlU/ml vs. 2,971 ± 4,780 mlU/ml, *p* = 0.27) or C5a (2,362 ± 4,529 mlU/ml vs. 2,971 ± 4,780 mlU/ml, *p* = 0.18) caused no significant increase of IFN-γ release.

**Table 3:** MT-specific IFN-γ releases of all performed MT IGRA test systems using co-stimulatory reagents

Assay	In-house MT IGRA^a^		EUROIMMUN Quan-T-Cell TB^a^		QuantiFERON^®^-Tb-Gold Plus^a^	
		IFN-γ [mIU/ml]		IFN-γ [mIU/ml]		IFN-γ [mIU/ml]
Antigen						
	TB10.4/ESAT6/CFP-10	2,971 ± 4,780	ESAT6/CFP-10 (TB-Mix)	2,548 ± 4,145	ESAT6/CFP-10 (Tube 1)	2,635 m ± 4,475
	+ CpG ODN	6,768 ± 21,097	+ CpG ODN	3,355 ± 5,425	+ CpG ODN	3,627 ± 5,992
	+ Poly(I: C)	2,578 ± 4,579	+ Poly(I: C)	2,633 ± 4,245	+ Poly(I: C)	1,973 ± 3,264
	+ C5a	2,362 ± 4,529	+ C5a	2,001 ± 3,553	+ C5a	2,343 ± 3,754
					ESAT6/CFP-10 (Tube 2)	2,759 ± 4,446
					+ CpG ODN	3,257 ± 5,349
					+ Poly(I: C)	2,518 ± 4,314
					+ C5a	36,770 ± 167,824
n^b^		25 of 71 total		24 of 71 total		24 of 71 total

^a^ The data are shown as means ± standard deviations (S.D.)

^b^ The amount of biomaterial per donor was not always sufficient to perform all stimulations which is why for some donors conditions had to be left out. This explains variations with regard to the group size (*n*)

**Fig. 3:**
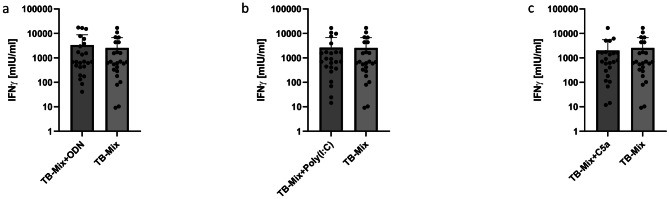
IFN-γ responses with EUROIMMUN assay (ESAT6/CFP-10) and costimulating agents. No significant increase in IFN-γ was seen with (**a**) TLR9 agonist CpG ODN, (**b**) Poly(I: C) and (**c**) C5a. All values are given as mean concentration mIU/ml ± S.D. Lower limit of detection (Background + 3x S.D.) was at 1.9–12.6 mIU/ml for IFN-γ. Paired student’s t-test. Following symbols pinpoint significant differences: * *p* < 0.05, ** *p* < 0.01, *** *p* < 0.001 (*n* = 24)

Regarding the prototypic EUROIMMUN Quan-T-Cell TB assay, there was no significant increase of IFN-γ responses after addition of costimulating factors to be found (Fig. 3; Table 3), although CpG ODN again trended towards higher IFN-γ values. The IFN-γ release reached 2,548 ± 4,145 mlU/ml without costimulation and 3,355 ± 5,425 mlU/ml with CpG ODN (*p* = 0.1), 2,633 ± 4,245 mlU/ml with Poly (I: C) (*p* = 0.63) as well as 2,001 ± 3,553 mlU/ml (*p* = 0.09) with C5a, respectively.

**Fig. 4:**
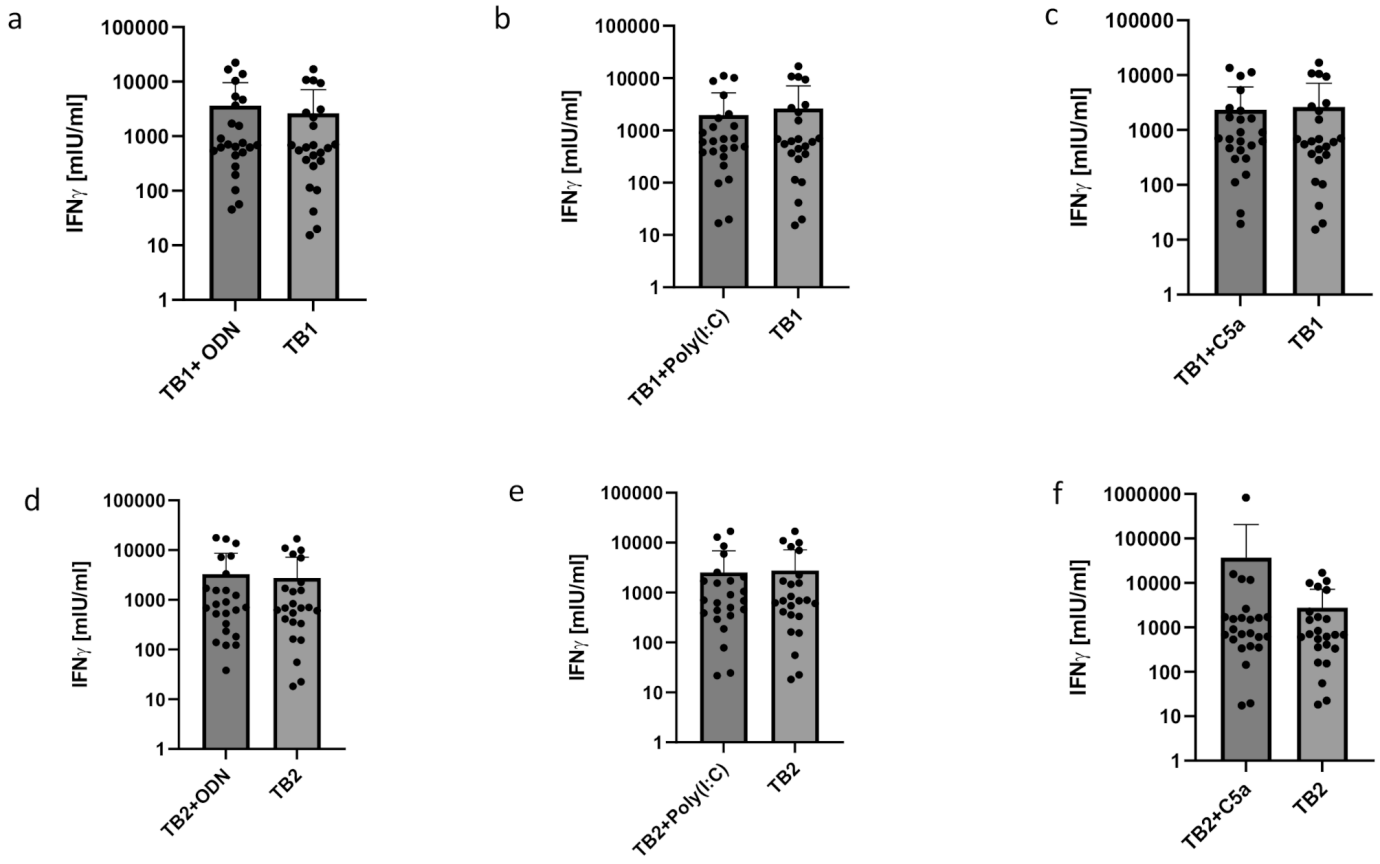
QuantiFERON^®^-Tb-Gold Plus tube 1 and tube 2 with costimulating agents. (**a + d**) CpG ODN, (b + e) Poly(I: C) and (**c + f**) C5a. All values are given as mean concentration mIU/ml ± S.D. Lower limit of detection (Background + 3x S.D.) was at 1.9–12.6 mIU/ml for IFN-γ. Paired student’s t-test. Following symbols pinpoint significant differences: * *p* < 0.05, ** *p* < 0.01, *** *p* < 0.001 (*n* = 24)

We also took the gold standard QuantiFERON Tb-Gold Plus assay into account (Fig. 4; Table 3). A trend towards increased IFN-γ responses was seen adding TLR9 agonist CpG ODN to tube 1 and tube 2 of the Quantiferon^®^-Tb-Gold-Plus test (3,627 ± 5,992 mlU/ml vs. 2,635 m ± 4,475 mlU/ml, *p* = 0.08, for tube 1; 3,257 ± 5,349 vs. 2,759 ± 4,446 mIU/ml, *p* = 0.25, for tube 2). No significant increase of IFN-γ release was seen when incubating tube 1 and tube 2 with Poly(I: C) or C5a. Tube 1 with Poly(I: C) reached 1,973 ± 3,264 mIU/ml vs. 2,635 ± 4,475 mIU/ml in tube 1 alone (*p* = 0.14), whereas tube 2 with Poly(I: C) was at 2,518 ± 4,314 mIU/ml vs. 2,759 ± 4,446 mIU/ml in tube 2 alone (*p* = 0.38). Tube 1 with C5a yielded 2,343 ± 3,754 mIU/ml vs. 2,635 ± 4,475 mIU/ml in tube 1 alone (*p* = 0.45), whereas tube 2 with C5a was at 36,770 ± 167,824 mIU/ml vs. 2,759 ± 4,446 mIU/ml in tube 2 alone (*p* = 0.38). Of note, the trend to remarkably mean higher IFN-γ responses within tube 2 plus C5a was due to a single outlier.

## Discussion

Our study aimed at improving the diagnostic performance of IGRAs for MT by including new antigens as well as introducing co-stimulatory reagents.

ACR, Rv1733 and Rv2626 failed to elicit equal or even better IFN-γ responses in our in-house IGRA compared to ESAT-6, CFP-10 and TB10.4 in whole blood of patients with TB disease.

The included control group consisting of patients with rheumatological underlying conditions showed significantly lower IFN-γ after stimulation with candidate and gold standard antigens compared to the patient group with tuberculosis disease.

Recent studies have shown the potential role for these novel antigens as immune dominant proteins in MT specific cellular immunity [21–24]. The groups of Geluk et al. and Arlehamn et al. showed that IFN-γ responses to ACR (also named HspX) were significantly higher in LTBI individuals than in MT-unexposed BCG vaccinees or healthy controls, respectively [21, 22]. Sera-Vidal and colleagues as well as Pena and colleagues published comparable data regarding the MT antigens Rv1733 and Rv2626c [23, 24]. Here, LTBI individuals secreted significantly higher IFN-γ levels against Rv1733 and Rv2626c than healthy controls. Of note, the studies with Rv1733 and Rv2626c reported a robust discrimination between patients with TB disease and LTBI individuals, with the latter yielding even higher positive IFN-γ responses than patients with active TB. All studies investigated MT-specific cellular immunity mostly using isolated leukocytes, i.e. PBMCs, in flow cytometry-based or enzyme-linked immunospot-based assays (ELISpot). Only Serra-Vidal et al. and Pena et al. used also an in-house IGRA and whole blood for the evaluation of Rv1733 as well as Rv2626c. Since the introduction of MT IGRAs over two decades ago there have been only a few selected studies addressing the identification of new immunodominant MT antigens representing a shortcoming in current basic as well as clinical research of tuberculosis.

To address the issue of co-stimulators in MT IGRAs we tested positive candidates from our earlier studies, i.e. the TLR agonists CpG ODN and Poly(I: C) and the complement factor C5a. The TLR agonist Poly(I: C) proved to increase IFN-γ responses in our CMV in-house IGRA [20], whereas CpG ODN and the complement factor C5a where effective in our HBV in-house IGRA [18, 19]. MT IGRAs represent another very promising test platform for the use of these co-stimulators in order to increase their diagnostic quality. CpG ODN delivered higher IFN-γ responses in whole blood of tuberculosis patients in the different IGRAs, albeit the effect did not reach statistical significance, compared to stimulation with MT antigens alone which may be attributed to the sample size (*n* = 71). The other two candidates Poly(I: C) and C5a did not show comparable effects. Further experiments are needed to determine why the selected co-stimulators appear to act pathogen-specific,, e.g. Poly(I: C) for CMV, C5a for HBV and CpG ODN for both HBV and MT. TLR agonists and complement factors target different receptors and signaling pathways in leukocytes. The G-protein coupled C5a receptor induces a different signal transduction cascade than the C3a receptor and both induce the expression of cytokines in T cells [26–28]. TLR agonists Poly(I: C) and CpG ODN affect cells of the innate as well as the adaptive immune system, i.e. increase transcription factor NFκB-dependent antigen processing and presentation on antigen presenting cells and induce higher cytokine expression in T cells [29–32].

There are some limitations to our study. One such limitation is the lack of an adequate group of LTBI patients which is due to the fact that Germany is a non-endemic country for MT. Another limitation concerns the evaluation of the three tested TLR agonists. They were not tested in combination which might have a synergistic effect on the stimulation and IFN-γ expression of MT-specific T cells in whole blood. Furthermore, TB disease was bacteriologically confirmed or clinically diagnosed. Our evaluation of the three novel antigens ACR, Rv1733 and Rv2626 might be somewhat affected negatively by a proportion of TB patients without clear bacterial confirmation in our cohort. Finally, the effects of anti-TB therapy as well as its duration on the strength of the IGRA results were not calculated because of the different therapy regimens used and the study design without follow-up visits.

## Conclusions

Taken together, in our study ACR, Rv1733 and Rv2626 did not yield strong and convincing enough IFN-γ responses in order to consider these antigens for use in MT IGRAs, whereas the TLR9 agonist CpG ODN might be useful as a co-stimulator which could be elucidated in further studies.

## Data Availability

The datasets used and/or analysed during the current study are available from the corresponding author on reasonable request.

## Abbreviations

BCG: Bacille Calmette-Guérin
CFP-10: 10 kDa culture filtrate protein
DMSO: Dimethyl sulfoxide
DosR: Dormancy survival regulator
ELISpot: Enzyme-linked immunospot-based assay
ESAT-6: 6 kDa early-secreted antigenic target protein
IGRA: Interferon gamma release assay
IVD: In vitro diagnostic
LTBI: Latent tuberculosis infection
MOTT: Mycobacteria other than tuberculosis
MT: Mycobacterium tuberculosis
PCR: Polymerase chain reaction
RR: Risk ratio
SEB: Staphylococcus aureus enterotoxin B superantigen
TB: Tuberculosis
TLR: Toll like receptor
TST: Tuberculin skin test

## References

[1] World Health O. Global tuberculosis report 2021. Geneva: World Health Organization; 2021 2021.

[2] Lonnroth K, Migliori GB, Abubakar I, D’Ambrosio L, de Vries G, Diel R, et al. Towards Tuberculosis elimination: an action framework for low-incidence countries. Eur Respir J. 2015;45(4):928–52. 25792630 10.1183/09031936.00214014 PMC4391660

[3] Houben RM, Dodd PJ. The global burden of latent tuberculosis infection: a re-estimation using Mathematical Modelling. PLoS Med. 2016;13(10):e1002152. 27780211 10.1371/journal.pmed.1002152 PMC5079585

[4] Long R, Divangahi M, Schwartzman K. Chapter 2: transmission and pathogenesis of tuberculosis. Can J Respiratory Crit Care Sleep Med. 2022;6(sup1):22–32.

[5] Getahun H, Matteelli A, Abubakar I, Aziz MA, Baddeley A, Barreira D, et al. Management of latent Mycobacterium tuberculosis infection: WHO guidelines for low tuberculosis burden countries. Eur Respir J. 2015;46(6):1563–76. 26405286 10.1183/13993003.01245-2015 PMC4664608

[6] Abubakar I, Drobniewski F, Southern J, Sitch AJ, Jackson C, Lipman M, et al. Prognostic value of interferon-gamma release assays and tuberculin skin test in predicting the development of active tuberculosis (UK PREDICT TB): a prospective cohort study. Lancet Infect Dis. 2018;18(10):1077–87. 30174209 10.1016/S1473-3099(18)30355-4 PMC6192014

[7] Tissot F, et al. Influence of bacille Calmette-Guérin vaccination on size of tuberculin skin test reaction: to what size? Clin Infect Dis. 2005;40(2):211–7. 15655737 10.1086/426434

[8] Trajman A, Steffen RE, Menzies D. Interferon-Gamma release assays versus tuberculin skin testing for the diagnosis of latent tuberculosis infection: an overview of the evidence. Pulm Med. 2013;2013:601737. 23476763 10.1155/2013/601737 PMC3582085

[9] Ho CS, Feng PI, Narita M, Stout JE, Chen M, Pascopella L, et al. Comparison of three tests for latent tuberculosis infection in high-risk people in the USA: an observational cohort study. Lancet Infect Dis. 2022;22(1):85–96. 34499863 10.1016/S1473-3099(21)00145-6 PMC8712384

[10] Streeton JA, Desem N, Jones SL. Sensitivity and specificity of a gamma interferon blood test for tuberculosis infection. Int J Tuberc Lung Dis. 1998;2(6):443–50. 9626600

[11] Qian F, Wang W, Qiu Z, Shen Y, He J, Li D, et al. Evaluation of a new tuberculosis-related interferon gamma release assay for tuberculosis infection diagnosis in Huzhou, eastern China. Indian J Pathol Microbiol. 2013;56(2):125–8. 24056648 10.4103/0377-4929.118694

[12] Della Bella C, Spinicci M, Alnwaisri HFM, Bartalesi F, Tapinassi S, Mencarini J, et al. LIOFeron(R)TB/LTBI: a novel and reliable test for LTBI and Tuberculosis. Int J Infect Dis. 2020;91:177–81. 31877486 10.1016/j.ijid.2019.12.012

[13] Kim JJ, Park Y, Choi D, Kim HS. Performance evaluation of a New Automated Chemiluminescent Immunoanalyzer-based Interferon-Gamma releasing assay AdvanSure I3 in comparison with the QuantiFERON-TB gold In-Tube assay. Ann Lab Med. 2020;40(1):33–9. 31432637 10.3343/alm.2020.40.1.33 PMC6713648

[14] Rangaka MX, Wilkinson KA, Glynn JR, Ling D, Menzies D, Mwansa-Kambafwile J, et al. Predictive value of interferon-gamma release assays for incident active tuberculosis: a systematic review and meta-analysis. Lancet Infect Dis. 2012;12(1):45–55. 21846592 10.1016/S1473-3099(11)70210-9 PMC3568693

[15] Zhou G, Luo Q, Luo S, Teng Z, Ji Z, Yang J, et al. Interferon-gamma release assays or tuberculin skin test for detection and management of latent tuberculosis infection: a systematic review and meta-analysis. Lancet Infect Dis. 2020;20(12):1457–69. 32673595 10.1016/S1473-3099(20)30276-0

[16] Oh CE, Ortiz-Brizuela E, Bastos ML, Menzies D. Comparing the diagnostic performance of QuantiFERON-TB Gold Plus to other tests of latent tuberculosis infection: a systematic review and Meta-analysis. Clin Infect Dis. 2021;73(5):e1116–25. 33289038 10.1093/cid/ciaa1822 PMC8423471

[17] Roupie V, Romano M, Zhang L, Korf H, Lin MY, Franken KL, et al. Immunogenicity of eight dormancy regulon-encoded proteins of Mycobacterium tuberculosis in DNA-vaccinated and tuberculosis-infected mice. Infect Immun. 2007;75(2):941–9. 17145953 10.1128/IAI.01137-06 PMC1828490

[18] Broker K, Terzenbach R, Bentzien F, Luth S, Dammermann W. Complement factors C3a and C5a mimick a proinflammatory microenvironment and increase HBV IGRA sensitivity. J Transl Med. 2019;17(1):6. 30602374 10.1186/s12967-018-1752-8 PMC6317231

[19] Dammermann W, Dornbrack J, Broker K, Bentzien F, Luth S. CpG oligonucleotides increase HBV-specific cytokine responses in whole blood and enhance cytokine release assay sensitivity. J Virol Methods. 2017;248:195–201. 28739303 10.1016/j.jviromet.2017.07.011

[20] Dammermann W, Bochmann D, Bentzien F, Komorowski L, Steinhagen K, Ullrich S, et al. CMV specific cytokine release assay in whole blood is optimized by combining synthetic CMV peptides and toll like receptor agonists. J Immunol Methods. 2014;414:82–90. 25450001 10.1016/j.jim.2014.10.011

[21] Geluk A, Lin MY, van Meijgaarden KE, Leyten EM, Franken KL, Ottenhoff TH, et al. T-cell recognition of the HspX protein of Mycobacterium tuberculosis correlates with latent M. Tuberculosis infection but not with M. Bovis BCG vaccination. Infect Immun. 2007;75(6):2914–21. 17387166 10.1128/IAI.01990-06 PMC1932904

[22] Arlehamn CS, Sidney J, Henderson R, Greenbaum JA, James EA, Moutaftsi M, et al. Dissecting mechanisms of immunodominance to the common tuberculosis antigens ESAT-6, CFP10, Rv2031c (hspX), Rv2654c (TB7.7), and Rv1038c (EsxJ). J Immunol. 2012;188(10):5020–31. 22504645 10.4049/jimmunol.1103556 PMC3345088

[23] Serra-Vidal MM, Latorre I, Franken KL, Diaz J, de Souza-Galvao ML, Casas I, et al. Immunogenicity of 60 novel latency-related antigens of Mycobacterium Tuberculosis. Front Microbiol. 2014;5:517. 25339944 10.3389/fmicb.2014.00517 PMC4189613

[24] Peña D, Rovetta AI, Del Hernández RE, Amiano NO, Pasquinelli V, Pellegrini JM, et al. A Mycobacterium tuberculosis Dormancy Antigen differentiates latently infected Bacillus Calmette–Guérin-vaccinated individuals. EBioMedicine. 2015;2(0):884–90. 26425695 10.1016/j.ebiom.2015.05.026 PMC4563115

[25] Dammermann W, Wollenberg L, Bentzien F, Lohse A, Luth S. Toll like receptor 2 agonists lipoteichoic acid and peptidoglycan are able to enhance antigen specific IFNgamma release in whole blood during recall antigen responses. J Immunol Methods. 2013;396(1–2):107–15. 23954282 10.1016/j.jim.2013.08.004

[26] Schraufstatter IU, Trieu K, Sikora L, Sriramarao P, DiScipio R. Complement c3a and c5a induce different signal transduction cascades in endothelial cells. J Immunol. 2002;169(4):2102–10. 12165538 10.4049/jimmunol.169.4.2102

[27] Arbore G, West EE, Spolski R, Robertson AA, Klos A, Rheinheimer C, et al. T helper 1 immunity requires complement-driven NLRP3 inflammasome activity in CD4(+) T cells. Science. 2016;352(6292):aad1210. 27313051 10.1126/science.aad1210 PMC5015487

[28] Liszewski MK, Kolev M, Le Friec G, Leung M, Bertram PG, Fara AF, et al. Intracellular complement activation sustains T cell homeostasis and mediates effector differentiation. Immunity. 2013;39(6):1143–57. 24315997 10.1016/j.immuni.2013.10.018 PMC3865363

[29] Drew PD, Franzoso G, Becker KG, Bours V, Carlson LM, Siebenlist U, et al. NF kappa B and interferon regulatory factor 1 physically interact and synergistically induce major histocompatibility class I gene expression. JInterferon Cytokine Res. 1995;15(12):1037–45. 8746784 10.1089/jir.1995.15.1037

[30] Lee KW, Lee Y, Kim DS, Kwon HJ. Direct role of NF-kappaB activation in toll-like receptor-triggered HLA-DRA expression. EurJImmunol. 2006;36(5):1254–66. 10.1002/eji.200535577 16619292

[31] Alexopoulou L, Holt AC, Medzhitov R, Flavell RA. Recognition of double-stranded RNA and activation of NF-kappaB by toll-like receptor 3. Nature. 2001;413(6857):732–8. 11607032 10.1038/35099560

[32] Kabelitz D. Expression and function of toll-like receptors in T lymphocytes. CurrOpinImmunol. 2007;19(1):39–45. 10.1016/j.coi.2006.11.007 17129718

